# Urgent care-seeking and injury severity for intimate partner violence during COVID-19: a Canadian retrospective chart review

**DOI:** 10.1186/s12889-023-16991-z

**Published:** 2023-11-06

**Authors:** Alison N. Ross, Emma Duchesne, Jane Lewis, Patrick A. Norman, Susan A. Bartels, Melanie Walker, Nicole Rocca

**Affiliations:** 1 Faculty of Health Sciences (School of Medicine), Queen’s University, Kingston, ON Canada; 2 Kingston Health Sciences Centre, 76 Stuart St, Victory 3, Kingston, ON K7L 2V7 Canada; 3 Department of Emergency Medicine, Queen’s University, Kingston, ON Canada; 4 Sexual Assault & Domestic Violence Program, Kingston Health Sciences Centre, Kingston, ON Canada; 5 Kingston General Health Research Institute, Kingston, ON Canada; 6 Department of Public Health Sciences, Queen’s University, Kingston, ON Canada

**Keywords:** Intimate partner violence, COVID-19 pandemic, Trauma, Injuries, Sexual assault, Domestic violence, Emergency department, Forensic medicine

## Abstract

**Background:**

Early reports raised alarms that intimate partner violence (IPV) increased during the COVID-19 pandemic, but initial studies showed that visits to emergency departments (EDs) decreased. This study assessed the impact of the prolonged pandemic and its associated restrictions on both rates of urgent care-seeking and injury severity for IPV.

**Methods:**

Data from the Kingston Health Sciences Centre’s (KHSC) ED were utilized to compare IPV presentations during ‘Pre-COVID’ (December 17, 2018 – March 16, 2020) and ‘COVID’ (March 17, 2020 – June 16, 2021), as well as three periods of heightened local restrictions: ‘Lockdown-1’ (March 17 – June 12, 2020), ‘Lockdown-2’ (December 26, 2020 – February 10, 2021) and ‘Lockdown-3’ (April 8 – June 2, 2021). The primary outcomes were incidence rate of IPV visits and injury severity, which was assessed using the *Clinical Injury Extent Score (CIES)* and *Injury Severity Score (ISS).*

**Results:**

A total of 128 individuals were included. This sample had mean age of 34 years, was comprised of mostly women (97%), and represented a variety of intimate relationship types. Some individuals presented multiple times, resulting in a total of 139 acute IPV presentations. The frequency of IPV visits during COVID was similar to the Pre-COVID time period (67 vs. 72; p = 0.67). Incidence rate was 13% higher during COVID, though this difference was non-significant (6.66 vs. 5.90; p = 0.47). IPV visit frequency varied across lockdown periods (11 in Lockdown-1, 12 in Lockdown-2 and 6 in Lockdown-3), with the highest incidence rate during Lockdown-2 (12.71). There were more moderate and severe injuries during COVID compared to Pre-COVID, but mean CIES was not statistically significantly different (1.91 vs. 1.69; p = 0.29), nor was mean ISS (11.88 vs. 12.52; p = 0.73).

**Conclusions:**

During the 15-months following the start of COVID-19, there were small, but non-significant increases in both incidence rate and severity of IPV presentations to the KHSC ED. This may reflect escalation of violence as pandemic restrictions persisted and requires further investigation.

## Background

Intimate partner violence (IPV) is a major public health problem globally. In Canada, as many as 44% of women and 36% of men experience some form of IPV within their lifetimes [1]. IPV carries substantial short- and long-term negative impacts for those who experience it, as well as their families and their communities more broadly [2–4]. Moreover, IPV is associated with significant morbidity and mortality [2, 5]. This ranges from exacerbation of physical and mental health conditions, to severe violence and death. In Canada, intimate partners kill approximately one woman every week [6]. This public health crisis seems to have escalated since March 2020 when the COVID-19 pandemic was first declared [7], based on numerous reports of increased IPV worldwide [8–13]. This “shadow pandemic”, as the United Nations has named it, has caused many organizations to call for more resources and research to be directed towards IPV [8, 9, 14].

In response to the pandemic, governments enacted a variety of policies in order to curb the spread of the virus, including quarantines, social isolation, travel restrictions and “stay-at-home” orders. Such restrictions fluctuated over time, often tightening in association with the emergence of new, more virulent variants [15, 16]. While such measures were necessary and effective for infection control, they led to significant social, economic and psychological disruption that may have inadvertently increased and exacerbated IPV [17, 18]. The situational factors that heighten risk for IPV, including increased social isolation, unemployment, greater exposure to a violent partner at home, increased substance use, and restricted access to public spaces, were largely increased with COVID-19 lockdown periods [18–22]. Amid lockdowns, many healthcare appointments shifted to phone and virtual formats, which often occurred in the home where patients lived with violent partners [21, 23]. This made it increasingly challenging to identify IPV in the outpatient setting and highlights the importance of the Emergency Department (ED) as a unique clinical environment to recognize IPV and offer specialized care.

Despite rising risk of violence, some studies showed that ED presentations for IPV decreased during the early pandemic [24, 25]. However, these studies did not evaluate data beyond June 2020, thus making the impact of prolonged and recurrent pandemic restrictions on urgent care-seeking for IPV largely unknown. Furthermore, no Canadian studies have assessed severity of IPV-associated injuries during COVID-19, despite studies showing an increase in IPV-related traumatic injuries internationally [24, 26, 27]. This study sought to fill these gaps by assessing how the COVID-19 pandemic and its associated restrictions affected the rate of presentation and injury severity for people experiencing IPV in the 15-months before and after the start of the pandemic in Kingston, Ontario, Canada.

## Methods

### Study design, setting and time period

This retrospective chart review study took place at the Kingston Health Sciences Centre (KHSC), a large academic tertiary care hospital located across two sites in Kingston, Ontario. KHSC provides complex, acute and specialty care for southeastern Ontario, a catchment area of more than 500,000 people. Data were obtained primarily from the KHSC’s Sexual Assault/Domestic Violence (SADV) Program, and were supplemented using ED (including Urgent Care Centre) charts and inpatient records (if applicable). The SADV is a specialized service within KHSC’s Department of Emergency Services and Ambulatory Care that offers 24/7 clinical care for people experiencing IPV and sexual assault (SA). It is also part of a broader network of centres (the *Ontario Network of Sexual Assault/Domestic Violence Treatment Centres*) offering comprehensive trauma-specific care and treatment. Patients of all ages and genders can access the program through self-referral, community-referral, or referral from a KHSC provider, typically an emergency physician.

The study timeline was based on the province of Ontario’s response to the pandemic [28]. On March 17th 2020, the province declared a state of emergency in response to the rising threat of COVID-19, which serves as the ‘pandemic start date’ for the purposes of this study. The primary study periods of interest consisted of ‘Pre-COVID’ (December 17, 2018 – March 16, 2020) and ‘COVID’ (March 17, 2020 – June 16, 2021). In order to also assess the impact of heightened restrictions on care-seeking, three COVID lockdown periods were identified, whereby stay-at-home orders and closures of most public establishments were in place regionally: ‘Lockdown-1’ (March 17 – June 12, 2020), ‘Lockdown-2’ (December 26, 2020 – February 10, 2021) and ‘Lockdown-3’ (April 8 – June 2, 2021).

### Population

This study included any adult patient who was seen by or referred to the KHSC SADV for IPV during the study periods. IPV was defined as any “behaviour by an intimate partner or ex-partner that causes physical, sexual or psychological harm”, typically as an attempt to assert power or control over the other [3, 29]. Patients who were identified as experiencing acute IPV, regardless of their original reason for presenting to care, were included. Additionally, those who consented to being referred to the SADV but subsequently declined care were included. Patients < 14 years of age (SADV’s ‘pediatric’ cut-off) and those seen for non-IPV reasons (e.g. SA by a stranger, family violence, etc.) were excluded.

### Data collection

Ethics approval was obtained from the Queen’s University Health Sciences Research Ethics Board (TRAQ#: 60333039) prior to data collection. The medical and SADV records from patients that met inclusion criteria were reviewed. All relevant sociodemographic details, presentation characteristics, assault-related information, medical management and forensic data were extracted by two chart reviewers (AR, JL). A third chart-reviewer (ED) audited 10% of charts for quality assurance.

Injury data were extracted from charts using free-text notation and *International Classification of Diseases-10 (ICD-10)* codes [30]. These data were scored by two independent raters who were blinded to the date of presentation. Injury severity was assessed using two tools: the *Clinical Injury Extent Score* (CIES, summarized in Table 1), and the *Injury Severity Score* (ISS, 0–75). The CIES is a simple, validated tool for the assessment of physical injuries in the context of IPV [31]. The ISS is an anatomic scoring system used widely in the setting of multi-system trauma, with scores ranging from 0 (no injuries) to 75 (unsurvivable injuries), and whereby “major trauma” is typically defined a ISS ≥ 16 [32–34]. To adequately capture the full spectrum of injuries, each presentation was initially graded with the CIES and those presentations deemed to be severe (CIES = 3) subsequently had an ISS calculated by an experienced data administrator. Inter-rater reliability for the CIES was calculated using κ-statistics and disagreements were settled by consensus.

**Table 1:** Clinical Injury Extent Score (CIES) Criteria†

Score	Criteria
0 (No Injury)	No documented signs or symptoms.
1 (Mild)	Redness or tenderness only or minor injuries with no expected effect on physical function.
2 (Moderate)	Injuries or injury expected to have some effect on function and/or more than redness-tenderness of the genitalia (including anal-rectal injuries; e.g. lacerations, bruising, abrasions) and/or injuries requiring treatment (lacerations requiring suturing, wounds requiring dressings) and/or bruising of the head and neck expected to result in significant headache.
3 (Severe)	Head injury with concussion and/or evidence of attempted strangulation and/or other major injuries (e.g. limb fracture, internal organ contusion).

^†^ CIES criteria created by McGregor et al., 1999

### Outcome measures

The primary outcome measures were: (1) frequency of IPV presentations, (2) incidence rate of IPV presentations, (3) CIES, and (4) ISS. These primary outcome measures were compared across the 15-months before and after the start of the pandemic, as well as across lockdown periods.

### Data analysis

Descriptive statistics included frequencies and percentages for categorical variables and means with 95% CIs for continuous variables. Characteristics of IPV presentations were analyzed between Pre-COVID and COVID using χ^2^-tests for categorical variables and *t*-tests for continuous variables (or Wilcoxon rank-sum tests for skewed data).

Frequency of IPV presentations was calculated using the total number of presentations to the SADV and total all-cause KHSC ED visits for the study population. These were compared using χ^2^-tests. The incidence rate of IPV presentations, expressed as a case-rate per 10,000 ED visits, was calculated by dividing the number of IPV visits in a given time-frame by the total number of ED visits during that same time-frame and multiplying by 10,000. Absolute differences were calculated by subtracting the pre-COVID values from the corresponding COVID values. The relative percentage change was calculated by dividing the absolute difference by the pre-COVID value, expressed as a percentage. Differences in injury severity were evaluated using mean CIES and ISS group scores and compared using *t*-tests and one-way ANOVA.

All tests were two-tailed and *p*-values < 0.05 were considered statistically significant. Data were analyzed using SPSS Ver-27, SAS Ver-9.4 (TS1M6) and SAS/STAT Ver-15.1.

## Results

A total of 128 individuals were included. The mean participant age was 34.1 years (SD = 11.1), 97% were women, and 45% had at least one child/dependant. IPV occurred across a variety of relationships, including dating-type relationships (45%), marriages/common-law (38%), and former relationships (17%). The sociodemographic characteristics of participants who presented during COVID were similar to those who presented during Pre-COVID (Table 2).

**Table 2:** Characteristics of the study population presenting to SADV for IPV before and during COVID-19 (N = 128)

Demographic Characteristics	Pre-COVID (Dec 17 2018–Mar 16 2020), *N = 69*, n (%)	COVID (Mar 17 2020-Jun 16 2021), *N = 59*, n (%)	*p*-value
Age (years)	33.0 (30.5–35.5) ^†^	35.5 (32.4–38.6) ^†^	0.19
Female vs. Male/Trans^§^	67 (97)	57 (97)	0.87
Relationship Status with Assailant			0.97
Marriage or Common-Law Intimate Relationship^‡^ Previous Intimate Relationship	27 (39) 30 (43) 12 (17)	22 (37) 27 (46) 10 (17)	
Number of Dependants			0.56
None One ≥ Two *Missing Data*	20 (29) 15 (22) 17 (25) *17 (25)*	13 (22) 17 (29) 11 (19) *18 (30)*	
Current or Previous Mental Health Diagnosis	48 (70)	40 (68)	0.83
Disclosed History of Abuse/Trauma	12 (17)	6 (10)	0.42
Current Homelessness or Housing Instability	4 (6)	8 (14)	0.35

Note: patients that presented multiple times during the study period are only represented once in this table (based on their first visit to care). Unless otherwise indicated, data in parentheses are percentages and *p*-values are calculated with Pearson χ^2^ tests.

^†^ Data are means with 95% confidence intervals and *p*-value was calculated using an independent sample t-test

^§^ Due to small cell sizes, males, trans and non-binary people are combined

^‡^ Includes any dating-type relationship (i.e. boyfriend, girlfriend, partner, etc.)

Of the 128 participants, eight presented more than once for acute IPV events during the study timeline (five presented twice, three presented three times). These repeat presentations were counted as unique IPV visits, resulting in a total of 139 IPV presentations. Table 3 summarizes the various characteristics of these visits. The majority of presentation and assault characteristics were similar across the two study periods. Many, and often multiple, forms of IPV were experienced, with 83% of events involving physical abuse, 19% involving sexual abuse, and 56% involving other forms of abuse (e.g. verbal, psychological, emotional or financial). Strangulation was reported in 28% of overall visits and 12% involved the use of a weapon (such as a knife, gun or other blunt force object). The most common location for an assault to have occurred was in a common/shared residence (43%). Approximately 31% of visits arrived by ambulance and only 27% of all visits had IPV (or equivalent) as the triage “reason for visit”. Major differences noted between the two time periods included more police involvement during COVID compared to Pre-COVID (66% vs. 53%; p = 0.03), IPV visits were generally triaged as less urgent (*Canadian Triage and Acuity Scale (CTAS)* Levels 3–5 vs. Levels 1–2; p = 0.01), fewer visits were mental health-related at triage (10% vs. 27%; p = 0.02), and fewer patients were accompanied by a visitor (16% vs. 43%; p < 0.01).

**Table 3:** Comparison of presentation, assault and management characteristics for IPV-related visits before and during COVID-19

Variables	Pre-COVID (Dec 17 2018–Mar 16 2020), *N = 72*, n (%)	COVID (Mar 17 2020–Jun 16 2021), *N = 67*, n (%)	*p*-value
**Presentation Characteristics**			
Time between Assault & Presentation to Care (days)	2.7 (0-5.4)^†^	2.1 (0.1–4.1)^†^	0.72
Accompanied by Someone to Hospital	31 (43)	11 (16)	< 0.01
Arrival by Ambulance	20 (28)	23 (34)	0.30
Seen in ED	64 (89)	60 (89)	0.90
Police Involvement	38 (53)	44 (66)	0.03
Child Welfare Involvement	20 (28)	19 (28)	0.26
**Assault Characteristics**			
Physical Abuse	59 (82)	56 (84)	0.80
Sexual Abuse	15 (21)	12 (18)	0.66
Other Forms of Abuse^§^	41 (57)	37 (55)	0.84
Strangulation	23 (32)	16 (24)	0.48
Assault with a Weapon	10 (14)	6 (9)	0.36
Location of Assault			0.26
Patient’s Own Residence Assailant’s Residence Shared/Common Residence Other (incl. public location, outdoors, etc.)	12 (17) 14 (19) 36 (50) 10 (14)	18 (27) 12 (18) 24 (36) 13 (19)	
Recent Life Changes/Stressors			
Separation or Change in Relationship Status Change/Loss of Employment Pregnancy or New Child (< 1yo)	17 (24) 5 (7) 7 (10)	25 (37) 8 (12) 6 (9)	0.08 0.31 0.88
**ED & Medical Management**			
CTAS^‡^			0.01
Level 1 or 2 (Resuscitation, Emergency) Level 3, 4 or 5 (Urgent, Less Urgent, Non-Urgent)	32 (50) 32 (50)	17 (28) 43 (72)	
Triage “Reason for Visit” was IPV (or equivalent)^‡^	20 (31)	18 (30)	0.88
Presented with a Mental Health Concern at Triage^‡^	17 (27)	6 (10)	0.02
Inpatient Admission	4 (6)	7 (10)	0.29
Specialist Referral(s) Required for IPV Injuries	11 (15)	16 (24)	0.20
**Injury Severity**			
CIES			0.15
0 (No Injuries) 1 (Mild) 2 (Moderate) 3 (Severe)	22 (31) 7 (10) 14 (19) 29 (40)	11 (16) 10 (15) 20 (30) 26 (39)	
**SADV/Forensic Management**			
Collection of Sexual Assault Evidence Kit (SAEK)*	2 (13)	2 (17)	0.99
Collection of Forensic Photography	28 (39)	23 (34)	0.68

Note: unless otherwise indicated, numbers represent frequencies, data in parentheses are percentages, and *p*-values are calculated with Pearson

χ^2^ tests. Some variables contain missing data points (< 5% of total sample), which were excluded from analyses.

^†^ Data are means with 95% confidence intervals and *p*-values are calculated using independent samples t-tests

^§^ Other forms of abuse included: verbal abuse, psychological abuse, emotional abuse, stalking, confinement/isolation, cyber-violence, destruction/theft of property, coercion, spiritual abuse, and financial abuse

^‡^ Data and *p*-values for these variables represent the sub-group of visits that were seen in the ED (n = 124) only

* Data and *p*-values for this variable represents the sub-group of visits that were associated with a sexual assault (n = 27) only

ED = Emergency Department | CTAS = Canadian Triage Acuity Scale | CIES = Clinical Injury Extent Score

Overall, there were 72 IPV visits during pre-COVID and 67 during COVID, an absolute decrease of 6.9% (p = 0.67). During this same period, all-cause ED visits to KHSC decreased by 17.6%, from 122,094 visits during Pre-COVID to 100,605 visits during COVID (p < 0.001). As shown in Table 4, IPV visits therefore made up a larger proportion of ED presentations during COVID (6.66 cases per 10,000 ED visits) when compared to Pre-COVID (5.90 cases per 10,000 ED visits), a clinically important but non-statistically significant increase of 12.9% (p = 0.47).

**Table 4:** Comparison of all-cause ED visits, SADV visits, and injury severity in Pre-COVID vs. COVID

	PRE-COVID	COVID	Abs. Difference	% Relative Change	*p*-value
**ED Visits**					
Total	122,094	100,605	-21,489	-17.6	< 0.01^†^
**SADV Visits**					
Total Case rate per 10,000 ED visits^§^	72 5.90 (4.61–7.42)	67 6.66 (5.16–8.46)	-5 + 0.76	-6.9 + 12.9	0.67^†^ 0.47
**Injury Severity**					
Mean CIES Scores^*^ Mean ISS Scores^*^	1.69 (1.39-2.00) 12.52 (10.33–14.70)	1.91 (1.64–2.18) 11.88 (8.74–15.02)	+ 0.22 -0.64	+ 13.0 -5.11	0.29 0.73

Note: the ‘Pre-COVID Period’ was December 17, 2018 – March 16, 2020 (456 days). The ‘COVID Period’ was March 17, 2020 – June 16, 2021 (456 days).

^†^*P*-values calculated using one-sample χ^2^ test with assumption of equal probabilities (since both time periods were of equal duration)

^§^ Case rates per 10,000 ED visits calculated using the number of ED visits in that time period as the denominator. Values in parentheses are 95% confidence intervals

* Data are means with 95% confidence intervals. *P*-values are calculated using independent samples t-tests

In terms of severity of IPV presentations, inter-rater reliability for CIES was strong (κ = 0.91, 95% CI 0.86–0.96) and Table 3 outlines the breakdown of scores across study periods. There were more moderate and severe injuries (CIES 2 and 3) during COVID (46/67 presentations) than during Pre-COVID (43/72 presentations). In turn, mean CIES was 13% higher during COVID (1.91; 95% CI 1.64–2.18) when compared to Pre-COVID (1.69; 95% CI 1.39-2.00), although not statistically significantly different (p = 0.29). Overall, a similar number of visits in each time period involved severe injuries (CIES = 3) – 26/67 presentations during COVID and 29/72 during Pre-COVID. Comparison of this sub-group did not reveal any significant differences in terms of mean ISS (12.52 in Pre-COVID vs. 11.88 in COVID; p = 0.73). Within this whole sample, ISS scores ranged from 1 to 26, and a total of 18 visits had scores of 16 or higher, therefore meeting criteria for *major trauma* [33, 34].

Table 5 summarizes data from each of the three lockdown periods. There were 11 IPV visits during Lockdown-1, 12 during Lockdown-2, and 6 during Lockdown-3 (p = 0.11). Notably, Lockdown-2 had the highest frequency of IPV visits, despite being the shortest in duration at 47 days. When compared to the entire Pre-COVID period, Lockdown-2 saw a 115.6% increase in the incidence rate of IPV visits per 10,000 ED visits (p = 0.012). There were no significant differences in terms of injury scores across lockdown periods, although Lockdown-1 had the highest mean CIES and ISS overall.

**Table 5:** Comparison of case presentations and injury severity during three COVID-19 lockdown periods

	Lockdown-1	Lockdown-2	Lockdown-3	*p*-value
Number of Days % of total COVID Time	88 19.3	47 10.3	56 12.3	
**ED Visits**				
Total	16,132	9,440	12,402	< 0.01^†^
**SADV Visits**				
Total Case-Rate per 10,000 ED visits^§^	11 6.82 (3.40–12.20)	12 12.71 (6.57–22.20)	6 4.84 (1.78–10.53)	0.11^†^ 0.10
**Injury Scores**				
Mean CIES* Mean ISS*	2.09 (1.39–2.79) 17.0 (7.63–26.37)	1.67 (0.80–2.54) 10.0 (0.03–19.97)	1.83 (0.29–3.38) 7.33 (0-34.57)	0.73 0.28

Note: Lockdown-1 was from March 17 – June 12 2020, Lockdown-2 was from December 26 2020 – February 10 2021 and Lockdown-3 was from April 8 – June 2 2021.

^†^*P*-value calculated using one-sample χ^2^ tests with the assumption of frequencies reflective of percent time within the total COVID period

^§^ Case rates per 10,000 ED visits calculated using the number of ED visits in that time period as the denominator. Values in parentheses are 95% confidence intervals. *P*-value is calculated using a χ^2^ test

* Data are means with 95% confidence intervals. *P*-values are calculated using a one-way ANOVA

## Discussion

This study was the first in Canada to assess prolonged pandemic restrictions on urgent care-seeking and injury severity for IPV. Findings showed that there was no change in the frequency of IPV visits during the 15-months following the start of COVID-19 restrictions in our region. However, there was a non-significant increase in the incidence of IPV visits when compared to all-cause ED visits during this time. Analysis of three time periods of heightened restrictions (“lockdowns”) showed that the proportion of IPV visits to the ED was highest during the second-wave of the pandemic (Lockdown 2: December 26, 2020 – February 10, 2021), and, to a lesser extent, the first-wave (Lockdown-1: March 17 – June 12, 2020). Evaluation of severity of IPV visits showed a non-significant increase in injuries during COVID compared to pre-COVID, with the highest degree of injury observed during the first wave of the pandemic (Lockdown-1).

These findings add to the growing body of literature assessing the impacts of the COVID-19 pandemic and its associated restrictions on IPV. While global data suggest that COVID-19 and its related policy response measures contributed to increases in IPV, clinical data have largely shown decreases in urgent care-seeking for IPV during the pandemic [10–13]. Both Muldoon et al. [25] and Gosangi et al. [24] found that rates of presentation to the ED for IPV decreased by around 50% during the initial phase of the pandemic (March–May 2020), compared to previous years. In contrast, we found that the absolute number of IPV visits during COVID was similar to that of Pre-COVID, and that relative rates showed a non-significant increase of 13%. This discrepancy may be related to our study’s longer timeline, potentially suggesting that initial decreases in urgent care-seeking for IPV were transient, and may have subsequently increased then eventually levelled-out to pre-pandemic rates over time. This is supported by Holland et al. [35], who found that while rates of IPV ED visits in the United States decreased during March 2020, they increased slightly from March–October of that same year. Subjective accounts from IPV service providers, including those working at shelters and crisis lines, also describe an initial decrease in contact volumes when COVID-19 lockdowns were first established, subsequently followed by an increase in volume after initial lifting of such restrictions [36]. An alternative explanation for this discrepancy could be related to the fact that Kingston and its surrounding area saw relatively low regional case-rates of COVID-19 during the first year of the pandemic [37, 38], which may have contributed to individuals feeling safer seeking-care at our ED compared to those in larger cities with higher COVID-19 transmission [39]. Given lower community transmission, local IPV services were largely able to remain operational throughout the pandemic, which was publicized through mass media campaigns [40]. These public outreach campaigns may also have contributed to more individuals seeking care in general, including in the ED, due to less confusion over what services were open, something previously cited as a barrier to accessing care and services during the pandemic [36].

With regards to injury severity, we did not find any significant difference between COVID and Pre-COVID. This is in contrast to Gosangi et al. [24] who reported more severe IPV-related injuries during the initial phase of COVID-19 (March 11 – May 3, 2020). Interestingly, this initial COVID period corresponds roughly to Lockdown-1 in our study, which was also the period with the highest average injury scores. While injury scores were not statistically different, we did observe a significant increase in police involvement during COVID, which is surprising given that previous literature has found police involvement to be a marker of more severe IPV [41, 42]. This incongruity may reflect that violence did in fact escalate over the course of the pandemic, but that our study was inadequately powered to detect it. Alternatively, it may be related to confounders unique to COVID-19, such as potentially more bystander intervention. For example, during stay-at-home orders neighbours may have witnessed/overheard IPV and reported it, resulting in earlier police involvement, de-escalation of violence and less severe injuries. Further, people experiencing IPV may have engaged police more often during COVID-19, given fewer safety options available to them amid increased strain on shelters [43, 44]. Other studies from different centres would be helpful to assess whether these data trends apply elsewhere. Regardless, we did find a relatively high severity of injuries across both study periods, with a max reported ISS of 26 – much higher than the max ISS of 10 reported by Gosangi et al. [24]. Further, 13% of the overall sample met criteria for major trauma and many individuals were strangulated and assaulted with weapons. This re-emphasizes the degree of morbidity associated with IPV and should serve as a reminder for emergency medicine practitioners to screen for and manage IPV appropriately, particularly given the potential for violence to escalate to fatal ends [45, 46].

The data presented herein have several important limitations. First, the study sample was relatively small, which made it difficult to compare trends in IPV-related care-seeking, particularly across lockdown-periods. Second, while injury scores are a helpful metric for injuries, they are a poor proxy for “severity” of IPV, as they do not reflect the many negative impacts of IPV beyond acute physical injuries. Finally, this study does not capture those who presented to the ED for IPV without disclosing to a care provider (which presumably could be quite numerous, given that less than 30% of visits disclosed IPV at triage), those who declined SADV engagement, those who sought care elsewhere, or those who did not seek healthcare at all. Therefore, these data have limited generalizability and should be considered an underestimate of the total number of individuals experiencing IPV in the community more broadly. Nevertheless, this study has various notable strengths, including the use of validated tools for the assessment of IPV injury severity, collection of comprehensive sociodemographic and assault characteristics from charts, use of blinding for subjective outcome measures (CIES and ISS), and high inter-rater agreement.

Overall, more studies are needed to assess the impact of the prolonged pandemic and its associated restrictions on urgent care-seeking for IPV, particularly in regions that initially saw decreases in IPV-related visits. Future research would benefit from qualitative data from patients to assess whether changes in community IPV services and outreach may have impacted the decision to seek care in the ED.

## Conclusion

This single-centre study from Kingston, Ontario, Canada showed that while there was no difference in the absolute number of IPV visits during COVID-19 compared to the 15-months prior, IPV did make up a higher proportion of ED visits during the pandemic, particularly during lockdowns. Additionally, there was a non-significant increase in IPV-related injury severity observed during COVID-19 that warrants further investigation.

## Data Availability

The data analysed in this study are not publicly available due to their sensitive nature. They are, however, available from the corresponding author (AR) on reasonable request.

## Abbreviations

IPV: Intimate Partner Violence
SADV: Sexual Assault/Domestic Violence Program
ED: Emergency Department
KHSC: Kingston Health Sciences Centre
CIES: Clinical Injury Extent Score
ISS: Injury Severity Score
CTAS: Canadian Triage Acuity Scale
SA: Sexual Assault
